# Supplementary material for: Perceived symptoms as the primary indicators for 30-day heart failure readmission
Source: PLoS One. 2022 May 5;17(5):e0267820. doi: 10.1371/journal.pone.0267820 (PMC9070923; doi:10.1371/journal.pone.0267820)
Supplement: Attachment — Submitted filename: ResponseToReviewers.docx [file pone.0267820.s002.docx]

Dear Editor and Reviewers,

Thank you kindly for your thorough review of the manuscript and for your comments and suggestions. We have revised the manuscript and believe it is strengthened after the opportunity to address your recommendations. We hope that our revisions are clear and acceptable to you. Thank you again.

| **From Editor** | **Response to Editor** |
| --- | --- |
| 1. Please ensure that your manuscript meets PLOS ONE's style requirements, including those for file naming. The PLOS ONE style templates can be found at  <https://journals.plos.org/plosone/s/file?id=wjVg/PLOSOne_formatting_sample_main_body.pdf> and  <https://journals.plos.org/plosone/s/file?id=ba62/PLOSOne_formatting_sample_title_authors_affiliations.pdf> | Thank you, we have reviewed these resources and updated accordingly. We have also updated the reference numbers in the text to the format of brackets [1]. |
| 2. Please ensure that you include a title page within your main document. We do appreciate that you have a title page document uploaded as a separate file, however, as per our author guidelines (<http://journals.plos.org/plosone/s/submission-guidelines#loc-title-page>) we do require this to be part of the manuscript file itself and not uploaded separately.  Could you therefore please include the title page into the beginning of your manuscript file itself, listing all authors and affiliations. | A title page was added at the beginning of the main document, the title page lists all authors and affiliations. |
| 3. In your Data Availability statement, you have not specified where the minimal data set underlying the results described in your manuscript can be found. PLOS defines a study's minimal data set as the underlying data used to reach the conclusions drawn in the manuscript and any additional data required to replicate the reported study findings in their entirety. All PLOS journals require that the minimal data set be made fully available. For more information about our data policy, please see <http://journals.plos.org/plosone/s/data-availability>.  "Upon re-submitting your revised manuscript, please upload your study’s minimal underlying data set as either Supporting Information files or to a stable, public repository and include the relevant URLs, DOIs, or accession numbers within your revised cover letter. For a list of acceptable repositories, please see <http://journals.plos.org/plosone/s/data-availability#loc-recommended-repositories>. Any potentially identifying patient information must be fully anonymized.  Important: If there are ethical or legal restrictions to sharing your data publicly, please explain these restrictions in detail. Please see our guidelines for more information on what we consider unacceptable restrictions to publicly sharing data: <http://journals.plos.org/plosone/s/data-availability#loc-unacceptable-data-access-restrictions>. Note that it is not acceptable for the authors to be the sole named individuals responsible for ensuring data access.  We will update your Data Availability statement to reflect the information you provide in your cover letter. | The minimal data set was obtained by the study statistician and will be uploaded as a Supporting Information file. The file is fully anonymized. |
| 4. Please ensure that you include a title page within your main document. You should list all authors and all affiliations as per our author instructions and clearly indicate the corresponding author. | A title page was added at the beginning of the main document, the title page lists all authors and affiliations, and clearly indicates the corresponding author. |
| **From Reviewer #1** | **Response to Reviewer #1** |
| 1. The gap in the existing literature, that this study seeks to fill, has not been appropriately highlighted. This should be done in the introduction section, to justify the need to conduct this study. Whilst sufficient background on the topic has been provided, the authors should clearly state whether studies similar to this one, evaluating self-reported symptoms, have been published, or if this is a fundamentally novel idea or implementation. | Yes, thank you for pointing this out. We have revised the last two sentences of the introduction  From: “Therefore, further research is critical to evaluate the post-hospital experience of patients with HF and the symptomatic characteristics related to rehospitalizations. This study aimed to evaluate patient responses to daily symptomatic, physiologic, and medication adherence characteristics and the relationship of these patient-centric experiences to 30-day HF post-hospitalization readmissions.”  To: “However, studies evaluating the subjective post-hospital experience of patient-reported symptoms to predict HF readmissions are lacking. This study addresses this gap by investigating daily symptomatic, physiologic, and medication adherence characteristics and the relationship of these patient-centric experiences to expand our understanding of factors contributing to 30-day HF post-hospitalization readmissions.” |
| 2. Clearly highlighting statistically significant outcomes in the tables, perhaps with an asterisk, would significantly improve readability and comprehensiveness of the data. | Statistically significant outcomes were highlighted on the tables with an asterisk. |
| 3. A clarifying statement about the spread of patients between HFrEF and HFpEF in this study would be appreciated. A stratification and comparison of data on these grounds or compelling reason against such an analysis could also be considered. | This is an important question, and based on our anecdotal experiences caring for patients we would agree that there are likely differences in the symptomatic experiences between HFrEF and HFpEF populations, for example more fatigue in HFpEF.  From the statistician: LVEF was included in the analyses as a continuous variable and was not statistically significantly related to any of the outcomes.  Dichotomizing as HFrEF and HFpEF would not result in anything different.  For example, comparing the two groups with respect to readmission within 30 days was not significant, p = 0.507.    This study also was not powered for specific sub-group analysis and there were uneven numbers in the HFrEF and HFpEF groups. We did have a robust discussion about this point and do believe this is an important consideration for future research with an intentional study design to answer the question. |
| 4. In lines 191-194, please remove the statements prefaced with the phrase “although not statistically significant”. Statistical non-significance fundamentally indicates the absence of compelling data to this end. Claiming a “higher hazard of readmission”, despite statistical non-significance, is erroneous. | Understood, this sentence has been deleted. |
| 5. In lines 214-219, the authors highlight relationships which have been associated with rehospitalization in previous studies but were not demonstrably prominent in these results. Please consider adding a statement also suggesting that these findings may be a results of the small sample size of this study and that further research and data would be needed to this end to provide a definitive conclusion. | This was revised,  From: “This may represent incomplete symptom evaluations in the prior studies, or some specific characteristics in this sample population who were primarily obese African American women with non-ischemic cardiomyopathies resulting in HFrEF.”  To: “This may be due to the small sample size in this study or specific characteristics in this sample population who were primarily obese African American women with non-ischemic cardiomyopathies resulting in HFrEF. This inconsistent finding may also represent incomplete symptom evaluations in prior studies.”  We did include the small sample size in the limitations section and the section of future research. |
| 6. In lines 252-253 the authors state that “this study affirmed that those patients who reported adherence with medications had fewer breathing problems, reported feeling less tired and were less likely to be readmitted.” This finding, as presented in Table 3 and Q9, presented with statistical non-significance. As mentioned above, providing statements about increased or decreased likelihood despite statistical non-significance is erroneous. Please remove this statement. | This statement was removed. |
